# The cost-effectiveness of home phototherapy for hyperbilirubinemia in neonates: results from a randomized controlled trial

**DOI:** 10.1038/s41598-023-37684-y

**Published:** 2023-07-03

**Authors:** Miriam Pettersson, Linda Ryen, Mats Eriksson, Andreas Ohlin

**Affiliations:** 1 Department of Paediatrics, Faculty of Medicine and Health, Örebro University, 701 82 Örebro, Sweden; 2 Faculty of Medicine and Health, School of Medical Sciences, Örebro University, Örebro, Sweden; 3 University Health Care Research Center, Faculty of Medicine and Health, Örebro University, Örebro, Sweden; 4 Faculty of Medicine and Health, School of Health Sciences, Örebro University, Örebro, Sweden

## Abstract

This study aimed to establish the cost-effectiveness of home phototherapy versus hospital phototherapy treating hyperbilirubinemia in neonates more than 36 weeks. Based on clinical results from a randomised controlled trial showing that home phototherapy for hyperbilirubinemia in term neonates is as effective as hospital phototherapy, we performed a cost-minimisation analysis to identify the most cost-effective alternative. We included costs for health care resource use as well as costs for transportation in connection with re-visits. The cost per patient was €337 for home phototherapy compared with €1156 for the hospital alternative indicating average cost savings of €819 (95% confidence interval €613–1025) or 71% per patient. Transportation and outpatient costs were higher in the home treatment group and hospital care costs were higher in the hospital group. Sensitivity analysis shows that results are robust also when allowing for uncertainty. Home phototherapy for neonates more than 36 weeks costs less than in-hospital phototherapy while being equally effective, meaning that home phototherapy is a cost-effective alternative to hospital treatment for infants with neonatal hyperbilirubinemia.

*Trial registration* NCT03536078. Date of registration: 24/05/2018.

## Introduction

Neonatal hyperbilirubinemia is an extremely common condition that affects around 80% of all newborn babies, with 2% to 3% requiring phototherapy to reduce their bilirubin^1,2^. Historically, phototherapy was performed only in hospital, but studies since the 1980s have reported on the effectiveness of at-home treatments^3,4^. Such treatments continue to accelerate with the development of fiber optic devices that are more easily transported than traditional overhead lamps^5^.

Several studies and meta-analyses have evaluated home phototherapy and shown it to be not only safe and effective, but also beneficial to both mother–infant bonding and patient satisfaction^6–14^. The latest American Academy of Pediatrics guidelines have therefore recently recommended home phototherapy as an alternative to in-hospital treatment^15^. Many authors have suggested that this practice will result in lower costs and create more available cots in neonatal intensive care units and maternal units when patients with jaundice no longer need to stay in hospital^11,12,16,17^. However, since studies with control groups are lacking, no high quality data exist to suggest how substantial this cost reduction could be^14^. We therefore conducted a randomized controlled trial, where results on safety and feasibility, stress and bonding and parental experiences previously has been published^6,7,18^. The aim of this study was to use the prospectively collected costs from the RCT to enable a health economic analysis.

## Methods

This study included newborn infants who were part of a multicenter randomized trial conducted from August 2016 to September 2019. The ethical review board in Uppsala, Sweden, approved the study (D 2015/226) and it was registered at clinicaltrials.gov 24/05/2018 (NCT03536078). The original dataset included 147 patients originating from six different Swedish hospitals, but to ensure the highest level of cost control, this analysis is limited to the 92 patients included at Örebro University Hospital. The inclusion criteria were a chronological age of more than 48 h, a gestational age above 36 + 0 weeks and a TSB above 18 mg/dl (300 µmol/L) between 48–72 h of age or TSB above 20.5 mg/dl (350 µmol/L) after 72 h of age. The exclusion criteria were blood group incompatibility, TSB at inclusion above 24 mg/dl (400 µmol/L), asphyxia, weight loss of more than 10%, an ongoing infection or any other severe illness.

**Figure 1:**
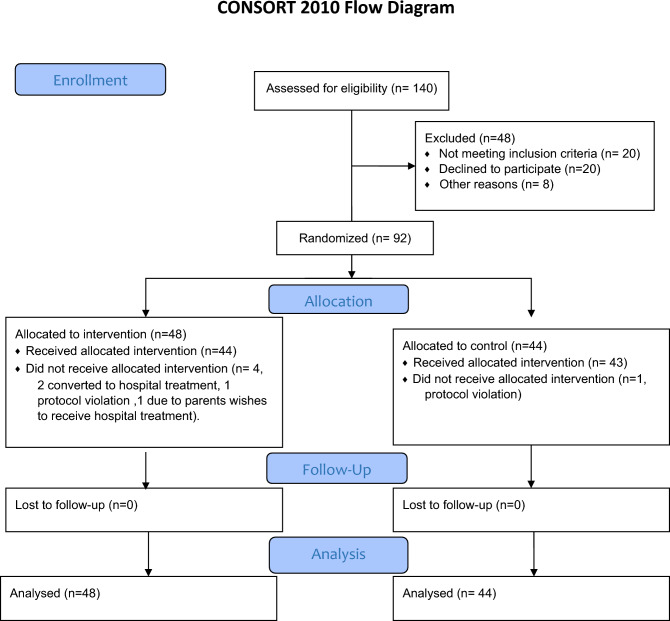
CONSORT Flow diagram.

After we received informed consent from parents, all patients were randomized in a block-wise design to either phototherapy at home using the Bilisoft fiber optic device (GE healthcare, Chicago IL, USA) or to standard in-hospital phototherapy. Details of the inclusion process can be seen in the CONSORT flow diagram (Fig. 1). The families randomized to home treatment were sent home but returned to the hospital once daily for bilirubin measurements and weight checkups. The admitted patients received all their phototherapy in hospital. They were sent home when their bilirubin levels fell below the treatment threshold, but returned for daily return visits for bilirubin and weight checkups, which explains why both groups had costs for transportation and outpatient visits. Both groups were discharged when their bilirubin levels dropped spontaneously without phototherapy. The clinical outcomes such as safety, feasibility, bonding between parents and infant, and breastfeeding have been extensively published in three earlier publications^6,7,18^.

### Health economic evaluation method

In a cost-effectiveness analysis, the result is traditionally presented in terms of the incremental cost-effectiveness ratio (ICER). For the strategies being compared or evaluated, the ICER estimates the difference in costs (measured in monetary units) divided by the difference in effects, thus expressing the cost of achieving one more unit of the effect. In this analysis, time until success of treatment was measured as time from first to last test. Since there was no significant difference in duration of phototherapy^6^, a cost-minimization analysis was performed for the base case. That is, when two strategies are equally effective, the one incurring the lowest cost is the cost-effective choice.

We performed sensitivity analysis by bootstrapping to acknowledge sampling uncertainty in both costs and effects. This procedure accounts for the variance in the study data by drawing repeated random samples with replacement of costs and effects from the intervention and control groups, with a sample size corresponding to the original sample. We drew 1000 sample pairs and estimated the average costs and effects for each. We also performed a deterministic sensitivity analysis, varying one variable while keeping the other constant, which allowed for uncertainty in cost estimations while facilitating generalizability to settings other than Sweden.

### Data on costs

Recently updated standards recommend that health economic evaluations present results from both a societal and a health care perspective^19^. In general, costs for in- and out-patient care, laboratory tests, and equipment are more related to the health care perspective and transportation costs to the societal perspective. However, in the Swedish setting, transportation costs for traveling to and from hospital are compensated by the healthcare system, so all costs reported in this study can be considered representative of the health care perspective.

Time and materials costs were based on actual resource use as registered in the study and monetarily valued mainly based on regional price tariffs. Details are presented in Table 1 and described below. All costs are stated in 2022 prices. The European Central Bank average rate for January 2022 was used to convert SEK to Euro (€1 = SEK 10.3579)^20^.

**Table 1:** Unit costs.

	Unit cost for estimation	Reference/comment
Day of inpatient care (neonatal department)	€1524	Administrative sources
Day of inpatient care (maternity care department)	€725	Administrative sources
Outpatient visit	€70	Administrative sources (assuming 15 min doctor/45 min nurse time)
Blood sampling		Administrative sources
Blood gas	€2.4	
Laboratory	€1.1	
Materials (cover, Bilisoft)	€4.2	Actual costs reported in the study
Transportation	€0.2 per km	Marginal cost for travel by car, based on subsidy offered by health care

No discounting was applied due to the short-term character of the intervention.

For both groups, the costs include hospital care days, outpatient re-visits, blood sampling, and consumables needed to use the Bilisoft equipment. We assumed no extra cost for the phototherapy equipment, as both groups used the same equipment for the same duration of treatment. Number of hospital care days is a measurement of the actual time spent in hospital. Length of stay was defined as time from the first to the last bilirubin test meaning that both in hospital and outpatient care is included. Duration of phototherapy was the actual time the patient spent on phototherapy. In this study, all families were admitted to a family room in the neonatal unit, but since phototherapy patients often are cared for in the maternity unit, we present estimates for both of these costs in the results.

Transportation distance was based on the distance between the hospital and the families’ postcode. All families were assumed to travel by car. The number of round trips was registered in the study for both groups; the marginal cost per km traveled was based on the compensation offered by healthcare for car travel to and from the hospital for treatment. This might underestimate the real cost, as the full cost might not be covered. However, not all of those included in the study would have traveled by car as some lived near the hospital. Also, cost and expenses are not equal: fuel taxes are only transferred within the society and should therefore be excluded.

No production loss was assumed, since both parents are allowed to claim compensation for caring for a sick child during treatment. This applies to both hospital and in-home phototherapy. Furthermore, no value or cost was assumed for time since there is no empirical evidence on the relative value of time spent at home, in hospital, or in transportation.

### Ethics approval

This study was performed in line with the principles of the Declaration of Helsinki. Approval was granted by the regional ethical review board in Uppsala, Sweden (2015/336).

### Consent to participate

Written informed consent was obtained from the parents.

### Consent for publication

Written informed consent was obtained from the parents.

## Results

Background characteristics of all included patients are presented in Table 2, and estimated costs for the two groups are presented in Table 3. The main result of the study is that one home treatment costs €337 compared with €1156 for the hospital alternative, for a cost reduction of €819 per session for each patient receiving home phototherapy. The costs for transportation and outpatient visits were higher in the home treatment group, while costs for hospital care were higher in the hospital group. Four patients originally allocated to the home group were subsequently admitted to hospital due to treatment failure or patient requests. These patients were analyzed according to intention-to-treat, meaning that patients allocated to home treatment that were readmitted to hospital resulted in a cost for in-patient care. This cost corresponded to an average cost of €91 per patient for inpatient care in the maternity unit.

**Table 2:** Characteristics of the study group.

	Home phototherapy (n = 48)	Hospital phototherapy (n = 44)
Gestational age (weeks), mean (SD)	39.3 (1)	38.9 (1)
Age at inclusion, days, mean (SD)	4.0 (1)	4.0 (1)
Birthweight (grams), mean (SD)	3545 (580)	3540 (506)
Serum bilirubin at inclusion, µmol/L, mean (SD)	362 (19)	360 (13)
Duration of phototherapy, hours, mean (SD)	22 (12)	23 (14)
Length of stay, hours	98 (51)	95 (70)

**Table 3:** Results from the health economic analysis.

	Home phototherapy Mean (SD) (n = 48)	Hospital phototherapy Mean (SD) (n = 44)	Mean difference (CI 95%) (statistically significant results in bold)
Number of days first to last bilirubin test	4.0 (2.1)	(2.9)	0.1 (− 0.9–1.2)
Number of hospital care days	0.1 (0.4)	1.4 (0.8)	− **1.3 (**− **1.5 to **− **1.0)**
Number of outpatient visits	3.0 (1.4)	1.8^a^ (1.5)	**1.2 (0.6–1.8)**
Number of blood samples	4.2 (1.4)	4.2 (1.9)	0.0 (− 0.7–0.6)
Bloodgas	4.1 (1.5)	4.2 (1.8)	− 0.1 (− 0.8–0.6))
Laboratory	0.1 (0.4)	0.1 (0.3)	0.1 (− 0.1–0.2)
Number of trips	6.1 (2.7)	3.6 (3.0)	**2.5 (1.3–3.6)**
Distance (km) from home to hospital^b^	15.6 (16.8)	16.3 (19.4)	− 0.7 (− 8.2–6.8)
Total transportation distance (km)	95 (115)	55 (72)	40 (− 0.1–80.0)
Estimated costs per infant			
Hospital cost neonatal unit	€191 (599)	€2113 (1282)	**€-1922 (**− **2331 to **− **1513)**
Hospital cost maternal unit	€91 (285)	€1005 (610)	**€-914 (**− **1109 to **− **720)**
Outpatient visits	€213 (95)	€127 (103)	**€86 (45–127)**
Materials	€4.1 (0.6)	€2.5 (2.1)	**€1.6 (1.0–2.3)**
Blood samples	€9.9 (3.5)	€10.1(4.4)	€-0.2 (− 1.8–1.5)
Transportation	€19 (22.9)	€11 (14.3)	€8.0 (− 0.2–16.0)
Sum of costs per infant			
Neonatal unit	€436 (606)	€2264 (1313)	**€-1827 (**− **2245 to **− **1409)**
Maternity unit	€337 (302)	€1156 (645)	**€-819 (**− **1025 to **− **613)**

^a^Estimated by number of round trips.

^b^Estimated by average distance to hospital from home postal code area.

### Sensitivity analysis

In the sensitivity analysis by bootstrapping, all 1000 drawings showed home phototherapy as the cost-saving alternative, with savings ranging from €483 to €1152 when hospital treatment was offered at the maternity unit.

For treatment duration, 40% of the drawings showed home phototherapy as the more effective, i.e. having a shorter treatment duration; however, the 60% of drawings with shorter hospital than at-home treatments showed that each reduced hour cost on average €600 when infants were treated at the maternity unit (implying a higher cost at the neonatal unit). In 65% of the drawings, the difference in treatment duration was ± 12 h. Fewer than 5% of the drawings showed differences > 24 h.

Deterministic analysis shows that transportation costs would need to be multiplied by 100 to change the recommendation by making hospital treatment at the maternity unit less expensive than treatment at home. If treating infants at the neonatal unit, transportation costs would have to be multiplied by more than a factor of 200 to be less expensive than treatment at home. Furthermore, outpatients visits would have to cost 10 times more per infant treated for home phototherapy to equal the cost of hospital treatment at the maternity unit, and more than 20 times higher to be as expensive as treatment at the neonatal unit.

## Discussion

Economic evaluations compare the cost and effects of possible interventions to inform decision-makers about how to use resources cost-effectively, which allows more health delivery within any given budget^21^. Home phototherapy has already compared favorably with in-hospital treatment on several factors. Although cost reduction might not be the decisive reason to start a home phototherapy program, evaluating the costs of new treatments is inherent to the research and very important in any administrative change in healthcare policy. In this paper we presented the first prospective health economic analysis of a home phototherapy program comparing costs with a randomly selected control group. The results show a substantial cost reduction of €819 (71%) per patient, mainly due to decreased costs for inpatient care, which accords with earlier reports on home phototherapy^8^. Sensitivity analysis shows robust support for the base case results**,** indicating cost savings for home treatment with no significant difference in effectiveness between treatment at home and in hospital.

In this study all families in the intervention group cared for their babies at home but returned to the hospital for all check-ups; no in-home visits were offered. This is probably why the cost reduction was so significant; many other home phototherapy programs offer at-home check-ups, which would naturally affect the result. Assuming the number of home visits would equal the number of outpatient visits for the intervention group while keeping costs for the control group constant, each home visit including costs for staff time and transportation would have to cost about €350 to match the costs of treatment at the maternity unit. This is based on the assumption that transportations costs would be the same as for the families, although healthcare practitioners may be able to cut costs by visiting more than one family before returning to the hospital.

Previous studies report high parent satisfaction with home phototherapy, which makes sense since presumably most people prefer the comfort of their own home to the environment of the hospital^9,14^. In our study the families in the home phototherapy group returned to the hospital every day and were offered no at-home visits. This obviously contributed to the high cost effectiveness of the program, but the home-treatment families also reported very positive experiences in a previously published qualitative study^18^. It seems the positive effect of avoiding a hospital stay is very strong. The European Association for Children in Hospital states in their first article that “children shall be admitted to hospital only if the care they require cannot be equally well provided at home or on a day basis”^22^.

Several aspects of treatment in the Swedish care setting may not be transferable to other countries, hence affecting the external validity of this study. Families in Sweden that are receiving phototherapy at hospital are frequently allowed leave to go home from the hospital. This means the control group would also have had transportation and outpatient visit costs, and their days to treatment completion would in many cases be higher than the number of their inpatient days. When a Swedish newborn infant needs hospital treatment, both parents are allowed to claim compensation for the care of their sick child, whether the child receives phototherapy at home or at the hospital. This means there is no between-group difference in production loss. In other countries, where different rules and cultural norms apply, production loss will vary depending on whether one or both of the parents returned to work during the phototherapy.

This study had a few limitations that we would like to point out. Even if the data originated from a multicenter study we only included the data from one center in the health economy analysis and only Swedish-speaking parents. A larger international multicenter design would have made the results more generalizable, but because the cost saving was so substantial, it is unlikely that the conclusion would change. Furthermore, the type of phototherapy (overhead lamp or fiber optic device) was not standardized for the in-hospital group, and this could have affected the effectiveness of the in-hospital therapy. This design was chosen, however, because we wanted to compare the intervention (home phototherapy) with the existing standard in-hospital therapy, which during the time of the study was a mix of overhead lamps and fiber optic devices. In addition, we did not record mode of transport or exact traveling distances for families’ hospital visits. Instead, when calculating traveling costs we used hospital to home postal code as a proxy for distance and assumed all travel was by car. This might have affected the precision of the calculations, but the randomized controlled design should ensure that there was no systematic between-group bias.

In conclusion, home phototherapy for neonates more than 36 weeks, including both healthcare and transportation, cost less than in-hospital phototherapy. Previously published results from this trial^6^ showed that home phototherapy, measured from first to last bilirubin test, was as effective as hospital. Therefore we conclude that home phototherapy is a cost-effective alternative to hospital treatment for infants with neonatal hyperbilirubinemia.

## Data Availability

The datasets used and/or analysed during the current study available from the corresponding author on reasonable request.
